# Immediate psychological outcomes associated with COVID-19 pandemic in frontline physicians: a cross-sectional study in Egypt

**DOI:** 10.1186/s12888-021-03225-y

**Published:** 2021-04-28

**Authors:** Mira Maged Abu-Elenin

**Affiliations:** Department of Public Health and Community Medicine, Faculty of Medicine,Medical Campus, Tanta University, 1st El-Geish Avenue, Tanta, 21561 Egypt

**Keywords:** COVID-19, Frontline physicians, Anxiety, Depression, HCWs, Mental health

## Abstract

**Background:**

The mental health of frontline healthcare workers is influenced by the crisis of the COVID-19 pandemic. This adversely affects their clinical performance and productivity. Therefore, it is important to recognize levels of anxiety, depression and identify the contributing factors.

**Methods:**

A cross-sectional study recruited physicians working at frontline positions in university teaching and isolation hospitals in the Mid Delta Region of Egypt from April to May 2020. Data was collected through an electronic online survey. Anxiety and depression levels were assessed using General Anxiety Disorder-7 and Patient Health Questionnaire − 9 respectively.

**Results:**

The study included 237 physicians, their mean age was 38.2 ± 6.2 years and 58% of them were males. Overall, 78.9% and 43.8% of all participated physicians reported symptoms of anxiety and depression. 85% of respondents had children with a significant increase in the risk of anxiety (OR = 20.2). This study revealed that poor sleep quality, being a resident physician, disrupted social life, and stigma exposure due to COVID-19, were significant mediating factors for the observed anxiety (OR = 0.53,3.28,0.18,1.56 respectively) and depressive symptoms (OR = 0.51,1.39,0.56,1.9 respectively). However, working in isolation hospitals wasn’t a significant contributing factor.

**Conclusion:**

The frontline physicians experienced a high rate of mental symptoms during the COVID-19 pandemic. That requires prompt intervention, taking into consideration the underlying determinants.

## Background

The World Health Organization (WHO) declared the Novel Coronavirus disease (COVID-19) as an international public health emergency by the end of January 2020. It was reported for the first time, in China in December 2019, and then continued to spread all over the continents [1].

The rapid vast spread, of this infectious disease, resulted in global awareness, anxiety, and depression. All of which according to WHO, are natural psychological responses to randomly changing conditions [2]. The adverse psychological effects among the general population are expected to increase significantly due to the pandemic itself, as well as the continuous flow of information through various types of media [3].

Furthermore, the implemented mass quarantine and curfew by the nationwide lockdown policies could lead to anxiety, depression, and distress. That could be attributed to the threat of losing the job, social distancing, family separation, insufficient basic needs, and financial losses. In addition to, the fear of disease progression, and increased the risk of contracting COVID-19 infection [4, 5].

Healthcare workers (HCWs) are generally at risk for exposure to highly infectious agents during the provision of medical care for their patients. As well as, the potential of the biologically contaminated environment in the health facilities. The possibility of transmission of infectious pathogens to their families is an issue, that exaggerates the worry of health providers and negatively affects their mental well-being [6].

The distress caused by epidemics and outbreaks might extend for a prolonged time and could lead to post-traumatic distress syndrome and depression [7]. It was reported that health professionals enrolled during the epidemic of the severe acute respiratory syndrome (SARS) in 2002–2003, suffered from a remarkable degree of anxiety and distress as a consequence of strict control measures, continuous surveillance, and reporting to the health authorities. Besides, their exposure to the patients’ sufferings due to illness and death [8].

Moreover, job instability and prolonged working hours put them in continuous conflicts between professional and family roles. Therefore, HCWs are more likely to experience a variant degree of mental health problems [9]. A meta-analysis study concluded that at least one in five healthcare professionals report symptoms of depression and anxiety during the COVID-19 crisis [10].

The WHO has also issued specific psychosocial considerations for the growing stigma of COVID-19, since the stigmatized community tends to seek medical care late and conceal important medical history. This behavior, in turn, will increase the risk of disease transmission within the community [11].

The associated stigmatization towards the infected cases was evident since the outbreak of SARS in 2003 [12]. Healthcare providers (HCP), particularly physicians, provided medical care for SARS-infected patient were found to be more prone to such stigmatization [13]. Similarly, the COVID-19 outbreak may also give rise to stigmatizing factors like fear of isolation, racism, discrimination, and marginalization [4]. It is believed that stigma and fear of infectious diseases hinder HCWs at all categories from responding properly and aggravated the imposed physical and mental stress of health care providers [14].

On the 25th of July 2020, there have been 15,581,009 confirmed cases of COVID-19, including 635,173 deaths were reported to WHO [15]. In Egypt from Feb 14th to 25th July 2020, there have been 91,072 confirmed cases of COVID-19 with 4518 deaths. According to the Egyptian Medical syndicate report on 15th July more than 3000 COVID-19 infections and 112 deaths were reported between Egyptian physicians [16].

For all these reasons, the psychological issues resulted from caring for potentially contagious patients should be addressed. To the author’s knowledge, so far there has not been any related research that addressed mental health problems during the COVID-19 pandemic among HCWs in Egypt. Therefore, this study aimed to compare levels of anxiety, depression among physicians working at frontlines in Tanta University teaching hospitals (TUH) and physicians working at Isolation hospitals (IH) in Gharbia governorate, Egypt.

## Methods

### Study design and setting

A cross-sectional study was conducted over a period of 1 month, from April to May 2020 in the Gharbia governorate in the Middle Nile Delta region which has an average population of 17 million. Tanta University Hospitals are the major tertiary care teaching hospitals with 1932 beds; provide secondary and tertiary healthcare services for 10 million populations reside in the region.

At the early stage of the crisis, the Ministry of Health and Population in Egypt assigned three hospitals to be Isolation Hospitals providing medical care for COVID-19 patients in the region. These hospitals were Kafr El-Zayat General hospital and El-Mahalla El-Kubra Chest hospital which belong to the Ministry of Health and Population. Also, a separate hospital belongs to Tanta University teaching hospitals.

Participants were recruited using a convenience sampling technique, the study targeted to include all actively enrolled physicians in the frontlines at Tanta university hospitals (TUH) and the three nominated Isolation Hospitals (IH) during the study period. Out of 254 invited frontline physicians, 237 expressed their willingness to participate in the study, and the response rate was 93.36%.

Physicians were contacted by the author through email/or telephone and were invited to participate in the study. An electronic survey link using google survey format was sent to participants’ emails and cellphones.

The nature and purpose of the study were explored in detail at the beginning of the electronic survey. It included a statement that they can withdraw from the study at any stage of the survey. Information privacy and confidentiality were ensured. After acceptance, the respondents were asked to fulfill the questionnaire anonymously and they were able to ask questions via the provided Email address for the author.

### Tools of the study

**Fig. 1:**
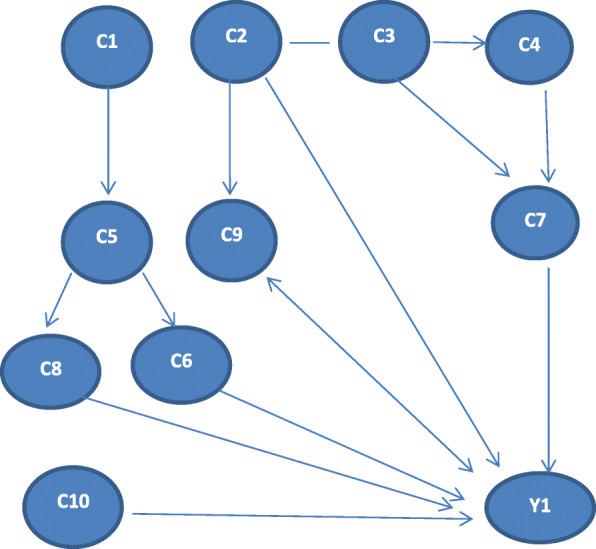
Directed acyclic graph of explanatory variables. C1: Age. C2: Gender. C3:Marital status. C4: Having children. C5: Professional degree. C6:COVID-19 related stigma. C7:Family support. C8:Sleep quality. C9:Psychological preparedness. C10:Material preparedness. Y: Outcome variable (mental health problems)

Data was collected by using an anonymous self-administered electronic questionnaire which consisted of four sections. The first one; included demographic characteristics, the effect of this pandemic on their personal life, and history of exposure to COVID-19 related stigma due to their profession. The selection of explanatory variables was mainly based on reviewing related published literature [1, 4, 5]. The theoretical rationale of selected explanatory variables is shown in Fig. 1.

The second section was related to mental health and preparedness data. Participants subjectively rated their psychological preparedness, material/supply preparedness, and sleep quality on a scale from 0 (not at all prepared/ sleep very poorly) to 10 (very well prepared /sleep very well). Material supply preparedness refers to; the availability of enough personal protective equipment, hand sensitizers, and medical supplies. Family support was also subjectively rated on a Likert scale of 0 (not at all supportive) to 4 (very supportive). For validation of this section, it was reviewed by three experts, whose notes and recommendations were taken into consideration (Cronbach α = 0.79).

The third section measured anxiety level using General Anxiety Disorder-7 (GAD-7) [17]. It had excellent internal consistency (Cronbach α = 0.92). Test-retest reliability was good (intra-class correlation = 0.84). Previous studies reported that Cronbach α was 0.89 for (GAD-7) psychometric tool [18, 19]. It consists of 7-items that asked respondents how they were bothered by each symptom often during the last 2 weeks. Response options were; “not at all, several days, more than half the days and nearly every day”, scored as 0, 1, 2, and 3, respectively. A cutoff score of ≥10 indicates anxiety disorder.

The fourth section assessed the degree of depression via the Patient Health Questionnaire (PHQ)-9 [20]. It is 9 items psychometric instrument, the scores for each depressive symptom are ranged from “0” (not at all) to “3” (nearly every day). Score ≥ 10 indicates moderate up to severe (major) depression.

In this study, the PHQ-9 test-retest reliability was also good (intra-class correlation = 0.82 and Cronbach α = 0.85. While previous reports showed a Cronbach α of 0.83 and 0.81 [21, 22].

### Statistical analysis

The organization, tabulation, and analysis of data were performed by using SPSS (IBM) Chicago version 21. Anxiety and depression were considered as the outcome variables. Explanatory variables included demographics, preparedness data, effects, and stigmatization due to COVID-19. Chi-squared test was used to examine the difference between substantial groups, when it was not appropriate, Fisher Exact test was applied instead. The probability of null hypotheses for the association between the outcome and independent variables was tested using Simple Logistic Regression analysis. The level of significance adopted was *p* < 0.05.

## Results

A total of 237 frontline physicians were included in the study, they were recruited from; Tanta University teaching hospitals (TUH) (*n* = 134) and the Isolation hospitals (IH) (*n* = 103). Baseline characteristics are shown in Table 1; the average age of participants was 38.2 ± 6.2 years ranged from 24 to 46 years. Male physicians constituted 58%, and mainly they were working at isolation hospitals 78.6% compared to 42.5% were in teaching hospitals. The majority were married and reported having children (about 85%). More than half of respondents were resident physicians (61.2%), with no distribution difference at both hospitals. About 41% of participated physicians experienced social stigmatization, more likely among physicians who were working at isolation hospitals (54.4%, *p* = 0.0002).

**Table 1:** Demographics and descriptive characteristics of participated physicians

	Total *n* = 237	TUH *n* = 134	IH *n* = 103	*Test of significance* *p-value*
	M (SD) or n (%)	M (SD) or n (%)	M (SD) or n (%)	
*Demographics*				
Age	38.2 ± 6.2	37.3 ± 6.7	39.2 ± 5.3	*t*-test = 2.36
				0.01*
Gender				
*Male*	138 (58.2%)	57(42.5%)	81 (78.6%)	X^2^ = 31.2
*Female*	99(41.8%)	77(57.5%)	22(21.4%)	< 0.0001***
Marital status				
*Single*	35(14.8%)	23(17.2%)	12(11.7%)	X^2^ = 1.41
*Married*	202(85.2%)	111(82.8%)	91(88.35%)	0.12
*Divorced /widowed*	0	0	0	
Having children				
*No*	34(14.3%)	20(14.9%)	14(13.6%)	X^2^ = 0.08
*Yes*	203(85.7%)	114(85.1%)	89(86.4%)	0.7
Professional degree				
*Registrar/ Consultant*	92(38.8%)	58(43.3%)	34(33%)	X^2^ = 2.58
*Residents*	145(61.2%)	76(56.7%)	69(67%)	0.1
COVID-19 related stigma				
*No*	140(59.1%)	93(69.4%)	47(45.6%)	X^2^ = 13.6
*Yes*	97(40.9%).	41(30.6%)	56(54.4%)	0.0002***
*Mental health and preparedness*	M (SD)	M (SD)	M (SD)	t-test *p*
Family Support	3.6 ± 1.3	3.6 ± 1.4	3.5 ± 1.3	0.56–0.7
Sleep quality	6.55 ± 3.2	6.5 ± 2.9	6.6 ± 2.7	0.27–0.7
Psychological preparedness	5.9 ± 3.2	5.7 ± 3.2	6.1 ± 3.2	0.95–0.3
Material preparedness	8.15 ± 1.8	8.1 ± 1.8	8.2 ± 1.85	0.41–0.6
GAD-7 scale	9.8 ± 4.7	10.25 ± 5.4	9.4 ± 5.2	1.47–0.007**
PHQ-9 scale	10.4 ± 3.6	10.4 ± 3.5	10.5 ± 3.9	0.2–0.9

df = 235 *TUH* Tanta University Hospitals, *IH* Isolation Hospitals

* *p* < 0.05, ** *p* < 0.01, ***p < 0.001

The mean rate of respondents for family support they received was fairly good (3.6 ± 1.3 out of 5). While the mean rate for sleep quality and psychological preparedness were poor; at 6.5 ± 3.2 and 5.9 ± 3.2 out of 10. However, the subjective rating for material preparedness was good 8.15 ± 1.8 out of 10. No statistical differences were detected regarding the respondents’ ratings at both hospital settings.

The overall mean perceived anxiety and depression scales were 9.8 ± 4.7 and 10.4 ± 3.6 respectively. Anxiety score symptoms were significantly higher among physicians working at university teaching hospitals 10.25 ± 5.4 versus 9.4 ± 5.2 among isolation hospitals’ physicians. No statistical difference was detected regarding depression score symptoms between physicians (Table 1).

**Fig. 2:**
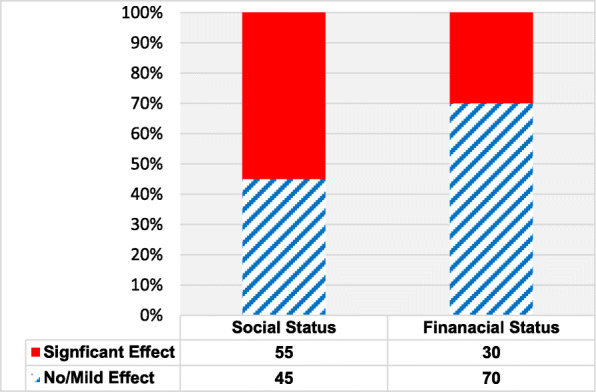
Perceived effect of COVID-19 Pandemic on social and financial life perspectives of participated physicians

About 30% and 55% of respondents reported that the pandemic had a negative consequence on their financial and social lives’ perspective as demonstrated in Fig. 2.

**Fig. 3:**
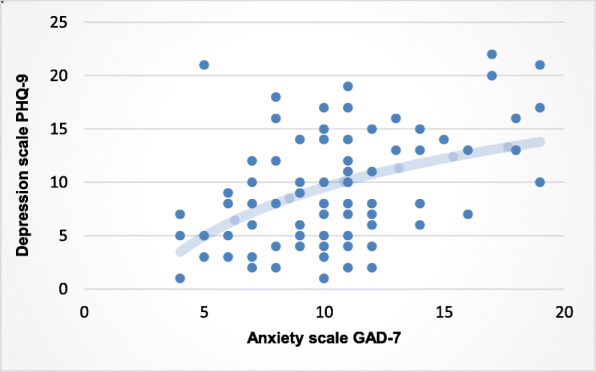
Correlation between depression and anxiety scales of participated physicians

A significant positive correlation was detected between the perceived depression scale (PHQ-9) and the perceived anxiety scale (GAD-7) (Pearson X^2^ = 4.29, r- coefficient = 0.48, *P* < 0.00001). Figure 3.

Table 2 demonstrated the logistic regression analysis of independents variables to the outcomes. It unveiled that, poor sleep quality, lack of psychological preparedness, having children, professional degree, affected social life and stigma exposure significantly and directly increased the odds of elevated anxiety symptoms (OR = 0.53,0.39,20.2, 3.28,0.18,1.56 respectively).

**Table 2:** Prevalence and correlates of anxiety and depressive symptoms among participated physicians

Independent Variable	Observed Anxiety Symptoms (GAD7 score ≥ 10) *n = 187*					Observed Depressive Symptoms (PHQ9 score ≥ 10) *n = 104*				
	***M (SD) n(%)***	B	S.E.	Wald	***Adj.OR(95%CI)***	***M (SD) n(%)***	B	S.E.	Wald	***Adj.OR(95%CI)***
**Age**	38.5 ± 5.9	−0.018	0.055	0.104	1.03 (0.9–1.1)	37.7 *± 8.2*	−0.013	0.023	0.303	0.99(0.94–1.03)
**Family support**	2.4 ± 1.35	0.04	0.07	0.2	0.74(0.48–1.13)	2.2 ± 1.4	1.6	0.8	15.2	0.64*** (0.5–0.84)
**Sleep quality**	**6** **± 2.8**	0.753	0.203	13.74	0.53** (0.37–0.71)	**5.72** **± 2.9**	−0.176	0.052	11.23	0.51**(0.35–0.74)
**Material preparedness**	8 ± 1.7	0.013	0.76	0.006	0.98 (0.69–1.39)	7.8 ± 1.8	−0.166	0.078	4.6	0.84(0.7–1.02)
**Psychological preparedness**	**5.1** **±** **3.1**	1.08	0.24	21.06	0.39*** (0.25–0.6)	5.6 *±* 3.2	−0.02	0.045	0.192	0.88(0.9–1.08)
**Gender**										
*Male (n = 138)* ^*□*^	104(55.6%)	−0.360	0.688	0.274		56(53.8%)	−0.403	0.320	1.58	
*Female(n = 99)*	83(44.4%)				2.74(0.69–10.7)	48(46.2%)				1.5(0.8–2.9)
**Marital Status**										
*Single (n = 35)* ^*□*^	27(14.4%)	−3.312	1.41	5.52		16(15.4%)	0.069	0.497	0.019	
*Married (n = 202)*	160(85.6%)				0.24(0.03–1.98)	88(84.6%)				0.97(0.35–2.68)
**Having Children**										
*No (n = 34)* ^*□*^	**28(15%)**	4.86	1.33	13.29		23(22.1%)	0.022	0.424	0.003	
*Yes(n = 203)*	**159(85%)**				20.2*** (3.7–108.4)	81(77.9%)				0.98(0.41–2.3)
**Working hospitals**										
*Teaching (n = 134)* ^*□*^	107(57.2%)	0.228	0.681	0.112		40(38.5%)	0.149	0.314	0.224	
*Isolation (n = 103)*	80(42.8%)				1.32(0.35–4.9)	64(61.5%)				0.8(0.43–1.5)
**Professional degree**										
*Registrar/Consultant*(*n* = 92) ^□^	**75(40.1%)**	0.85	0.273	15.6		**36(34.6%)**	−0.18	0.072	9.231	
*Residents* (*n* = 145)	**112(59.9%)**				3.28* (3.60–11.5)	**68(65.4%)**				1.39*(1.1–1.74)
**Affected social life**										
*No and mild effect (n = 107)* ^*□*^	**93(49.7%)**	−1.48	0.69	4.59		**53(51%)**	0.447	0.286	2.45	
*Significant effect (n = 130)*	**94(50.3%)**				0.18 * (0.04–7.8)	**51(49%)**				0.56*(0.5–1.01)
**Affected financial Status**										
*No and mild effect (n = 166)* ^*□*^	128(68.4%)	1.054	0.76	1.89		70(67.3%)	−0.14	0.322	0.191	
*Significant effect (n = 71)*	59(31.6%)				3.01 (0.73–12.3)	34(32.7%)				1.23(0.64–2.37)
**Experience social Stigmatization**										
*No(n = 140)* ^*□*^	**123(65.8%)**					**26(25%)**				
*Yes(n = 97)*	**64(34.2%)**	1.6	0.89	10.7	1.56** (1.24–1.97)	**78(75%)**	1.2	0.9	18.05	1.9*** (1.42–2.57)

Bold font indicates significantly different prevalence and odds ratios (*p* < 0.05)

^□^ Referent group * *p* < 0.05, ** *p* < 0.01, ***p < 0.001

Concerning depressive symptoms, it was found that lack of family support, poor sleep quality, professional degree, affected social life, and stigma exposure were significant independent variables for the increased risk of observed depressive symptoms among the studied physicians (OR = 0.64, 0.51,1.39,0.56 and 1.9 respectively).

## Discussion

The critical role of HCWs during pandemics is a vital substantial. They are susceptible to mental health problems due to the overwhelmed work and the massive fear of contracting an infection [23]. Significant short and long-term psychological effects on HCWs in the frontline positions were proved during the past outbreaks and epidemics such as SARS-cov-1, H1N1 influenza, and Ebola virus [24, 25].

This cross-sectional study enrolled 237 frontline physicians during the COVID-19 pandemic, the gender distribution showed that 58% of them were male with a significant male predominance among physicians of isolation hospitals 78.6%(*n* = 81). It is thought that more male physicians were assigned or volunteered to work at isolation hospitals than female physicians. Otherwise, there wasn’t any demographic difference between physicians who worked in both hospital settings.

The anxiety score was significantly higher among physicians who worked at University teaching hospitals (10.25 ± 5.4) than their colleagues at isolation hospitals (9.4 ± 5.2). This is probably because physicians at university hospitals are dealing with all patients in general without knowing about their actual infectious status. That made them more worried, suspicious, and cautious rather than providing medical care to confirmed COVID-19 patients. On the other hand, depression scores were relatively high among physicians at both hospital settings (10.4 ± 3.5 and 10.5 ± 3.9 respectively).

Noteworthy, more than half of physicians in this study suffered from disturbed social life probably due to time constraints, prolonged working hours, and abstinence from home. Meanwhile, 30%of them reported major concerns about the adverse effect of COVID-19 on their financial status probably due to the applied lockdown measures [26].

The contagion of infection makes HCWs more prone to emotional distress, which hinders their clinical roles and professional practices [27]. As well, it might lead to frustration and helplessness, which render them tremendous mental health stress during the ongoing COVID-19 crisis [28].

At the early stage of the COVID-19 pandemic, the infection rate among HCWs constituted about 29% of all hospitalized COVID-19 patients [29]. Recently a published article reported that infection was detected in 6.4% of HCWs in the Netherlands; to the incidence of 38.9% in China [30]. Meanwhile, a much lower rate was reported by the participated physicians in the present study 2.5% (*n* = 6). This discrepancy could be attributed to the enrollment of all categories of healthcare providers in the previous studies.

The present study revealed a significant prevalence of mental health symptoms among the respondents; overall 78.9 and 43.8% of all participated physicians reported symptoms of anxiety and depression respectively. Which is considered higher than those in the published studies worldwide; in Germany 14.5, 2.2%, in Singapore 8.1,10.9%, in Italy 16.6, 20.3% and in China 44.6, 50.4% [31–34].

A meta-analysis study calculated a pooled prevalence rates for anxiety and depression between HCWs during the COVID-19 crisis of 23.2 and 22.8% respectively [10]. These figures were similar to the respective rates among the general population in China, which ranged between 22.6 to 36.3% for anxiety and 16.5 to 48.3% for depression. That indicates the considerable effect of the crisis on the whole of the population [29, 35].

At the regional level, a study conducted in Saudi Arabia detected mild, moderate, and high anxiety scores at 68.25, 20.8, and 10% respectively among HCWs due to COVID-19 crisis [5].

Our figures were as high as reported in a previous study during the acute SARS outbreak, where 89%of health care workers who were engaged, experienced psychological symptoms [35]. Meanwhile, our figures were not similar to the reported rates in response to previous pandemics; a study in Greece found that nearly half of HCWs experienced moderate to high levels of worry during the H1N1 influenza pandemic [36]. Besides, more than half of HCWs in Japan and Singapore showed a high level of fear and anxiety during SARS-CoV outbreaks [37, 38].

Thought out that the variability of levels of mental symptoms among HCWs regarding infectious disease pandemics relies on several determinants, including study design, the psychometric measuring tool, as well as the timing of the study pre- or post- outbreak [5].

Of note that the detected level of anxiety symptoms in the current study was consistent with a previous study investigated stress level among resident physicians at (TUH). It revealed that 60.8 and 37.8% of studied residents had a moderate and high degree of job stress [26]. That indicates the pre-existence of the underlying contributing factors which are aggravated due to this emergent pandemic.

In the present study, logistic regression analysis was conducted to investigate the antecedents lying beneath the observed anxiety and depression symptoms experienced by the studied physicians. It revealed no gender difference regarding the rate of anxiety and depression. Though, similar literature expressed being a female was associated with experiencing more depression, anxiety, and distress [5, 34, 39].

Frontline medical staff, with a lack of PPE or other essential supplies, is more common to fear about their safety [29]. Most of the respondent physicians in the present study rated the material preparedness in their related hospitals to be very well prepared. It was found that material preparedness was not an associated factor for the observed mental symptom. Likely, was a study held in Wuhan [39].

The present study didn’t prove that working at isolation hospitals could implicate the observed anxiety and depression among physicians. This observation reflects that being in the frontline position itself is considered as a predisposing factor for the elicited mental symptoms irrespective of the nature of the healthcare facility.

Poor sleep quality was associated with both elevated anxiety and depression symptoms (OR = 0.53,0.51 respectively), which is supported by similar studies in Italy (OR = 0.53, 0.49 respectively) and in Wuhan (OR = 1.26, 1.31 respectively) [31, 39].

Anxiety symptoms were significantly associated with low psychological preparedness (OR = 0.39, 95% CI = 0.25–0.6). This could be attributed to the reduced accessibility to formal psychological support during this pandemic [32] This observation is consistent with the observed anxiety among HCWs in a similar study in China (OR = 1.18, 95% CI = 0/97–1.45) [39]. These findings replicate those from a study during the avian influenza pandemic in the United Kingdom, where two-thirds of doctors felt they were not ready. They also, reported that they were not well psychologically prepared and they lacked the proper support in such an experience [40].

The previous observation might be related to the explosive onset of the pandemic with a progressing influx of suspected and confirmed cases of COVID-19 to healthcare facilities. Which imposed pressures upon frontline HCWs, and less first-hand medical information about this outbreak [1, 32]. Another explanation is that when doctors are more professionally and psychologically trained for emergencies and managing isolated patients, they will have a lower level of anxiety and a higher resilience with a positive attitude [40].

The logistic regression results also showed that lack of family support has been associated with depressive symptoms with odds of 0.64 compared to Du et al. 2020, who detected an Odds of 2.4 for insufficient family support with increased depressive symptoms [39]. In contrast, a study reported that social support was negatively correlated with anxiety and stress in China [41].

Some literature that explored factors related to HCWs’ psychological conditions, had recognized that fear of infection of family members is the major concern of HCWs in COVID-19 affected areas [25, 42]. That explains why the presence of children significantly increased the odds of suffering from anxiety by 20 times among the studied physicians.

Furthermore, mental symptoms were more common among resident physicians than registrar and consultant physicians where they had an increased odd of 3.28,1.39 times for anxiety and depression respectively. This finding is consistent with a recent study that demonstrated that HCWs in frontline line positions were more likely to experience anxiety and depression OR = 1.57 (1.22–2.02) and 1.52 (1.11–2.09) [34]. The same was reported in a previous study conducted during the period of the MERS-CoV epidemic [43].

The previous observation could be attributed to the fact that resident physicians face patients all the time. Besides their closer contact with patients, they are more prone to moral harm about patients’ sufferings, illness, death, and an ethical dilemma [10].

Sources of stress during epidemics may include feelings of vulnerability, concerns about the health of self, and spreading the infecting virus to family members [44]. The before-mentioned reasons, in turn, explain how the affected physicians’ social status significantly had odds of 0.18 and 0.56 for both the increased risk of anxiety and depression. The affected financial status didn’t show any significant contribution to the measured mental symptoms.

It was reported that stigmatization against HCWs was associated with increased psychological distress and physical symptoms [45]. It is worthy to note that more than one-third of participated physicians experienced COVID-19 related stigma, particularly those who worked at isolation hospitals (54.3%) compared to their colleagues at University teaching hospitals (30.6%) (*p* = 0.0002).

The present study found that exposure to stigma had an odds ratio of 1.56 and 1.9 for anxiety and depression respectively. Likely, Ramaci et al.2020 concluded that stigma exposure positively predicted burnout and fatigue. While, it negatively predicted satisfaction among HCWs during the COVID-19 pandemic (OR = 0.24, 0.3, − 0.3 respectively) [14].

## Conclusion

Frontline physicians who responded to the spread of COVID-19 in Egypt had reported high rates of anxiety and depression symptoms irrelative to their working settings. The study highlighted that lack of family support, poor sleep quality, low psychological preparedness, having children, residency period, disrupted social life, and exposure to COVID-19 related stigma were significant antecedents for the observed mental symptoms.

### Recommendation

Our findings suggest that frontline physicians should be closely monitored as a high-risk group for depression and anxiety. On-going provision of psychological support and immediate intervention to HCWs who are exposed to the COVID-19 pandemic may promote their resilience to stress and enhance their mental wellbeing.

### Study limitation

First, data obtained from a self-administered questionnaire and data related to preparedness, sleep quality, and family support was based on self-report. Second, the study was carried out for 1 month and lacked longitudinal follow-up for the long-term psychological implications among this susceptible population. The small sample size of the study is attributed to the implemented quarantine and social distancing measures. Finally, the study didn’t assess the socioeconomic status which may help to evaluate the possible association with the outcomes and tailing proper intervention programs.

## Data Availability

The datasets generated and/or analyzed during the current study are not publicly available due to the relatedness of the recruited data to Tanta university hospitals and the local health authority in the Gharbia governorate, but are available from the corresponding author on reasonable request.

## Abbreviations

HCWs: Healthcare workers
IH: Isolation Hospitals
TUH: Tanta University Hospitals
